# CD49b, a Major Marker of Regulatory T-Cells Type 1, Predicts the Response to Antiviral Therapy of Recurrent Hepatitis C after Liver Transplantation

**DOI:** 10.1155/2014/290878

**Published:** 2014-01-19

**Authors:** Stenard Fabien, Morales Olivier, Ghazal Khaldoun, Viallon Vivian, Aoudjehane Lynda, Ouaguia Laurissa, Goormachtigh Gautier, Calmus Yvon, Delhem Nadira, Conti Filomena

**Affiliations:** ^1^Centre de Transplantation Hépatique, Pôle Digestif, APHP, Hôpital Saint Antoine, 184 Rue de Faubourg Saint Antoine, 75012 Paris, France; ^2^UPMC Université Paris 6, Inserm, UMR_S938, Centre de Recherche Saint Antoine, 75012 Paris, France; ^3^UMR 8161, Institut de Biologie de Lille, 1 Rue du Professeur Calmette, 59028 Lille, France; ^4^Unité de Biostatistique, Hôpital Cochin, 27 Rue du Faubourg Saint-Jacques, 75014 Paris, France

## Abstract

The TRANSPEG study was a prospective study to assess the efficacy of antiviral therapy in patients with a recurrent hepatitis C virus (HCV) after liver transplantation. The influence of regulatory T-cells (Tregs) on the response to antiviral therapy was analyzed. Patients were considered as a function of their sustained virological response (SVR) at 18 months after treatment initiation. A transcriptomic analysis was performed to assess Treg markers (Tr1 and FoxP3^+^) in serum, PBMC, and liver biopsies. 100 patients had been included in the TRANSPEG study. Data from 27 of these patients were available. The results showed that the expression of CD49b (a predominant marker of Tr1) before the introduction of antiviral therapy was significantly associated with SVR. Responders displayed lower serum levels of CD49b than nonresponders (*P* < 0.02). These findings were confirmed in PBMC and liver biopsies even if in a nonsignificant manner for the limited number of samples. The assessment of CD49b levels is thus predictive of the response to antiviral therapy. This data suggests that CD49b may be a marker of the failure of the immune response and antiviral therapy during HCV recurrence. The assessment of CD49b could help to select patients who require earlier and more intensive antiviral therapy.

## 1. Introduction 

Cirrhosis due to hepatitis C virus (HCV) is becoming the main indication for liver transplantation (LT). Cumulative studies have revealed that HCV is not directly cytopathic to hepatocytes [1]. It has been demonstrated that T helper-1 lymphocyte (Th1) or cytotoxic T lymphocyte (CTL) responses are critically involved in HCV-mediated liver injury [2]. HCV infection of the graft is universal, and graft damage is often accelerated, leading to cirrhosis in 30% of patients within five years [3], and reduced patient survival compared to other indications [4, 5]. The mechanisms of accelerated HCV-induced liver damage after LT are poorly understood.

Viral clearance seems to be associated with persistent CD4^+^ and CD8^+^ antiviral responses [6]. Strong Th1 activity, specific to Core, NS3, NS4, and NS5 proteins, is associated with spontaneous recovery [7], whereas a weak or non-persistent CD4 response is associated with a poor outcome [8]. The role of CD8^+^ T cells during acute infection has been clearly demonstrated [9]. Much attention has recently focused on regulatory T-cells (Tregs) and their contribution to HCV disease. The classic CD25^+^ Treg population, which accounts for 5–10% of peripheral CD4^+^ T-cells, constitutively expresses CD25 [10] and can suppress host immune responses in the setting of autoimmune diseases, transplantation, and antitumour immunity [11, 12]. Treg also constitutively expresses surface markers such as the glucocorticoid-induced tumour necrosis factor receptor family-related Gene, GITR (CD133) [13], cytotoxic T-lymphocyte antigen 4 CTLA4 (CD152) [14], and the transcription factor Foxp3, which is characteristic of this subpopulation [15]. Other subpopulations of regulatory T-cell subsets, such as IL-10-secreting Tr-1 cells that express CD18 and CD49b [16, 17], and TGF-*β*-secreting Th3 cells [18] have also been described.

There is some evidence to suggest that pathogens can exploit Treg to create a favourable immunological environment in order to escape an adequate immune response. In chronic HCV infection, the activation of virus-specific T-cells may be suppressed by CD4^+^CD25^+^ Treg, which might contribute to the inadequate immune response to HCV. An increased frequency of CD4^+^CD25^+^ T cells was recently found in the blood of patients with persistent HCV infection, compared with those who had cleared HCV [19, 20]. The *in vitro* depletion of CD25^+^ T-cells results in increased HCV-specific T-cell responsiveness. It has been proposed that CD4^+^CD25^+^ cells contribute to HCV persistence by suppressing HCV-specific T-cell responses [21, 22]. Some studies have also shown a correlation between a reduced HCV-specific T-cell response and the secretion of TGF-*β* by liver-infiltrating CD4^+^CD25^+^ T-cells [19], and regulatory T-cells are able to inhibit HCV-specific T-cell activity, independently of IL-10 and TGF-*β* [20, 21]. However, it has also been shown that functional Foxp3^+^CD4^+^CD25^+^ Tregs are detectable during both persistent HCV infection and after recovery, suggesting that they are part of the normal immune response to this pathogen [22]. In chronically infected patients, IL-10 secreting Tr1 cells circulate concomitantly with interferon (IFN) *γ*-secreting Th1 cells, and these two subtypes of CD4 T-cells recognize the same epitopes on HCV core protein [23], suggesting that Tr1 cells may also be implicated in HCV pathogenesis. In the postorgan transplant setting, numerous experimental studies have demonstrated that Tregs induce allograft tolerance [24, 25]. Tregs are influenced by immunosuppressive therapy; in particular, calcineurin inhibitors reduce Treg function *in vitro* [26]. It has also been shown that Treg levels fall significantly after LT, especially during allograft rejection [16, 27], and that the reduction in circulating Tregs is counterbalanced by an intragraft accumulation [28]. We recently evaluated the expression and activity of regulatory T-cells and their potential involvement in the accelerated progression of recurrent hepatitis C after LT. Our results suggested that classical CD4^+^CD25^+^ Tregs were significantly enhanced in recurrent hepatitis C and that Tr1 cells were specifically enhanced in severe recurrent hepatitis C [29]. We also found that serum IL-10 levels, characteristic of the Tr1 subset, were enhanced in patients prior to a severe recurrence, when compared with patients experiencing a mild recurrence of HCV [29].

In the present study, we further analyzed the potential correlation between regulatory T-cells (and particularly Tr1 cells) and the response to antiviral therapy after LT. For the first time, we have shown that Tr1 levels, as evaluated by the expression of the CD49b surface marker in serum, PBMC, or liver before the introduction of antiviral therapy, were significantly lower in patients who would develop a sustained virological response (SVR). Since the determination of CD49b in serum seems to be predictive of treatment response, this new tool may be useful in the selection of liver transplant patients with HCV recurrence who are candidates for more intensive antiviral therapy.

## 2. Patients and Methods 

### 2.1. Patients

Our study population was a cohort of 27 patients who had been included in the TRANSPEG study, which was a prospective, multicentre study (15 French centres) to assess the efficacy of antiviral therapy after LT, designed to determine whether 52 weeks of ribavirin maintenance monotherapy following 52 weeks of combination therapy procured an additional beneficial effect in recurrent HCV infection [30]; 101 adult recipients were enrolled, and all had proven chronic hepatitis with detectable serum HCV. Patients had to receive a stable calcineurin inhibitors immunosuppressive regimen. The 27 patients of 2 centres were included in this ancillary study.

**Figure 1:**
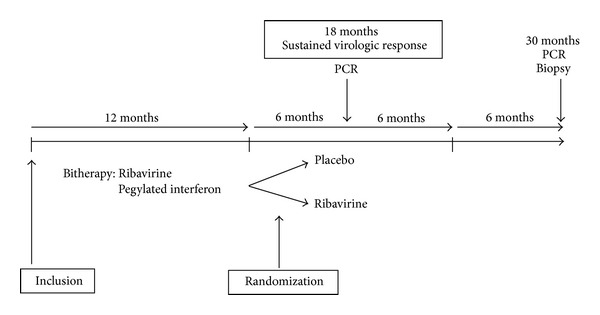
Design of the TRANSPEG study. All patients received Peginterferon alfa-2a and ribavirin. After 52 weeks of combination therapy, the patients were randomized to receive either ribavirin at the same dosage or a placebo for a further 52 weeks. All patients were followed up for 24 weeks after the end of treatment. The main time points in the analysis were: baseline, 52 weeks (end of combination therapy), 78 weeks (6 months after the end of combination therapy), and 130 weeks (end of follow-up). The primary efficacy endpoint was (1) sustained virological response, defined as undetectable serum HCV RNA. The secondary endpoints were changes from baseline histology findings as assessed by (2) the METAVIR activity and fibrosis score at 52, 78, and 130 weeks; (3) alanine aminotransferase (ALT) values over time; (4) the number of rejection episodes and the severity of rejection; (5) the occurrence of other severe antiviral-related adverse effects.

The patients received peginterferon alfa-2a and ribavirin; after 52 weeks patients were randomized to receive either ribavirin or a placebo for further 52 weeks. All patients were followed up for 24 weeks after the end of therapy (Figure 1). The main time points in the analysis were baseline, 52 weeks (end of combination therapy), 78 weeks (6 months after the end of combination therapy), and 130 weeks (end of followup). The primary efficacy endpoint was a sustained virological response (SVR), defined as undetectable serum HCV, 6 months after the end of therapy.

The TRANSPEG study and the present ancillary study were approved by the Ethics Committee (CCPPRB) of Rennes (France). All patients gave their written informed consent before undergoing any protocol-related procedures.

Serum samples, available only from 27 patients enrolled in the TRANSPEG study, were evaluated for Treg transcriptomic analysis, and peripheral blood mononuclear cells (PBMCs) and liver biopsies were also available from some of these patients.

### 2.2. Transcriptomic Analysis

Transcriptomic analysis was performed on serum samples, PBMCs, and biopsies; using this technique on a large panel of genes, we had previously found a correlation between the liver, PBMC, and serum regarding the transcription of 219 genes [31]. Serum samples were analyzed at baseline, before the beginning of the treatment, at 12 months (end of the course of ribavirin and pegylated interferon), at 18 months, and at 30 months (6 months after the end of second therapeutic cycle) (Figure 1).

### 2.3. RNA Extraction

Total RNA was extracted from frozen liver biopsy samples and PBMCs using the RNeasy midi kit (QIAGEN, Courtaboeuf, France), as described by the manufacturer. In summary, samples were first lysed and then homogenized. Ethanol was added to the lysate to provide ideal binding conditions. The lysate was then loaded onto the RNeasy silica membrane for RNA binding, and all contaminants were efficiently washed away. Pure, concentrated RNA was eluted in water and stored at −80°C.

Serum mRNA extraction was achieved using the QIAamp Viral RNA kit (QIAGEN, Courtaboeuf, France), as described by the manufacturer. Briefly, a serum sample was incubated with AVL buffer supplemented with Carrier RNA. After the addition of ethanol, the sample was applied to the QIAmp spin column and centrifuged. The column was then cleaned, and mRNA was eluted and stored at −80°C prior to analysis.

### 2.4. Reverse Transcription

RNA was supplemented with the following mixture [oligo dT (Roche, Meylan, France) + RNAsin (PROMEGA, Charbonnières; France) + H_2_O], and then incubated at 70°C for 5 minutes. After further 5 minutes at room temperature, the following mixture [buffer 5X (Invitrogen, Cergy-Pontoise, France) + DTT (Invitrogen) + dNTPs 10 mM + RNeasy (PROMEGA) + Superscript (Invitrogen)] was added. This reaction was followed by an initial incubation step at 45°C for 60 minutes and a second incubation at 95°C for 5 minutes. Finally, H_2_O was added to obtain a concentration of 10 ng total RNA/1 *μ*L.

### 2.5. ABI PRISM^R^ 7000 Sequence Detection System

The quantification of transcripts from liver, serum, and PBMC samples was performed using real-time quantitative RT-PCR with the ABI PRISM^R^ 7000 sequence detection system (Applied Biosystems, CA, USA). All primers were designed for real-time PCR (Table 1) and purchased from MWG-Biotech (Germany). The mix was optimized for real-time PCR analysis using SYBR Green 1 Dye, AmpliTaq Gold DNA polymerase, dNTPs with UTP, Passive Reference 1 required for signal normalization, and optimized buffer components, in a total volume of 50 *μ*L. Each sample was run in a 96-well plate containing 94 primers by performing initial denaturation at 95°C for 5 minutes, after which PCR reactions were cycled 40 times as follows: 15 seconds at 95°C and 1 minute at 60°C. Fluorescence intensity was measured at the end of each elongation phase. Melting curve analysis was performed immediately after amplification, in accordance with the manufacturer's instructions. The amplification of liver cDNA was successfully repeated twice with cDNA from the same extraction.

### 2.6. Light Cycler-Based PCR Assay

Genes whose expression level reached significance between the two groups of patients (SVR versus non-SVR) were further analyzed using light cycler-based PCR assay.

cDNA was synthesized from total RNA at a concentration of 100 ng/*μ*L using oligo dT primers and superscript reverse transcriptase (GIBCO BRL). The quantification of transcripts from samples was confirmed by real-time quantitative RT-PCR using the light cycler system (Roche Diagnostics, Meylan, France). The PCR mixture contained the following: *Taq* polymerase, 1X of LightCycler-DNA master SYBRGreen I (Roche Diagnostics), 3 mM MgCl_2_, 0.5 *μ*mol/L of each primer, and 1 *μ*L of the cDNA preparation (patient cDNA samples), in a total volume of 20 *μ*L. Thirty-two samples were run in parallel by performing an initial denaturation at 95°C for 8 minutes, and then the PCR reactions were cycled 35 to 40 times as follows: 15 seconds at 95°C, 7 seconds at the appropriate annealing temperature (Table 2), and 18 to 64 seconds at 72°C according to the length of the target sequence annealing (40 s at 58°C). Fluorescence intensity was measured at the end of each elongation phase. A melting curve analysis was performed immediately after amplification, following the manufacturer's instructions.

### 2.7. Primers

All primers were designed for real-time PCR use and were purchased from MWG-Biotech (Table 2). The housekeeping genes *β*-Actin, Ubiquitin, HPRT, and G3PDH were used as controls. Genes representative of the Treg population were chosen, such as CD4, a glycoprotein predominantly found on the surface of helper T-cells, CD25, the alpha subunit of Interleukin-2 receptor, FoxP3, a member of the forkhead/winged-helix family of transcriptional regulators [32], CD127, the interleukin 7 receptor [33] that is specific to naturally occurring Treg (CD4^+^CD25^high^CD127^−^Foxp3^+^), CD18, the integrin beta chain beta 2 [18], and CD49b, an integrin alpha subunit specific of Tr1 cells [34].

### 2.8. Cytometric Bead Array (CBA)

To detect cytokines in the serum samples from our patients, the BD CBA Human Th1/Th2/Th17 Cytokine Kit (Becton Dickinson, San Jose, CA) was used, as described by the manufacturer. The CBA assay permits simultaneous cytometric quantification of multiple cytokines in a sample by capturing them to spectrally distinct beads. Seven bead populations with distinct fluorescence intensities were coated with capture antibodies specific for IL-2, IL-4, IL-6, IL-10, TNF, IFN-*γ*, and IL-17A proteins. Human TH1/TH2/TH17 cytokine standards were reconstituted for 15 min in assay diluents, according to the manufacturer's immunofluorescence staining protocol. For all cytokine calibrator standards, serial dilutions were performed so that the serum was mixed with each human cytokine-capture bead combination, and then further incubated together with both the PE detection reagent and standard dilutions for 3 h at room temperature. The samples were then analyzed with a CANTO flow cytometer [35], using FCAP Array software to generate the results. Specific controls containing FITC- and PE-conjugated antibodies were supplied by the manufacturer [35] and were performed before each experiment.

### 2.9. Data Interpretation and Statistics

PCR results were analyzed using the “Relative Gene Expression Method,” as described elsewhere [33]. Briefly, individual CT values were normalized using the average CT values for housekeeping genes (ΔCT = CT − CT_HKG_). Average ΔCT values for each group were then compared with the ΔCT values of the control group (S group) using Wilcoxon-Mann-Whitney statistics. Statistical tests associated with *P* values lower than 0.05 were considered to be significant. For relevant genes, binary prognostic tests for the response to antiviral therapy were constructed. To do this, optimum cut-off values were determined according to Youden's index. The sensitivity and specificity of these tests were then computed. It should be recalled that in this setting, sensitivity was defined as the probability of a positive test at J0 for responders (at M18), while specificity was the probability of a negative test at J0 for nonresponders (at M18). Statistical analyses were performed using SAS software, version 9.1.

## 3. Results

### 3.1. Characteristics of the Study Population

The study cohort comprised 27 patients from the 101 patients included in the TRANSPEG study that was designed to evaluate antiviral therapy in recurrent hepatitis C. Serum samples for the Treg analysis, and clinical data collected during the TRANSPEG study, were available for all of these 27 patients. Table 2 shows the demographic parameters of this population of 20 men and 7 women, with a median age of 56 years. METAVIR scores for fibrosis were distributed as follows: *F*1 in 15 patients, *F*2 in 8 patients, and *F*3, *F*4 in 3 and 1 patients, respectively. The patients were split into two groups in terms of their response to the primary criteria of the Transpeg protocol, that is, their virological response at 18 and 30 months (M) after inclusion. 62% of the 27 patients available were virological responders at M18 and continued to display a virological response at M30 (Table 3). These rates were similar to those obtained for all the patients included in the Transpeg study. Because the SVR rates were identical at M18 and M30, we therefore considered SVR as the virological response obtained at M18 and analyzed the parameters relative to this response. Furthermore, as in the whole Transpeg study, maintenance therapy with ribavirin alone did not improve either the virological response or the histological parameters (data not shown). The characteristics of patients in both groups (responders and non-responders) at M18 are shown in Table 3, Viral load, METAVIR score for fibrosis, immunosuppressive therapy, and donor age were similar in the two groups.

### 3.2. Serum Gene Expression of T Regulatory Markers at Baseline

The serum expression of classic Treg markers was similar in patients with and without SVR (data not shown).

**Figure 2:**
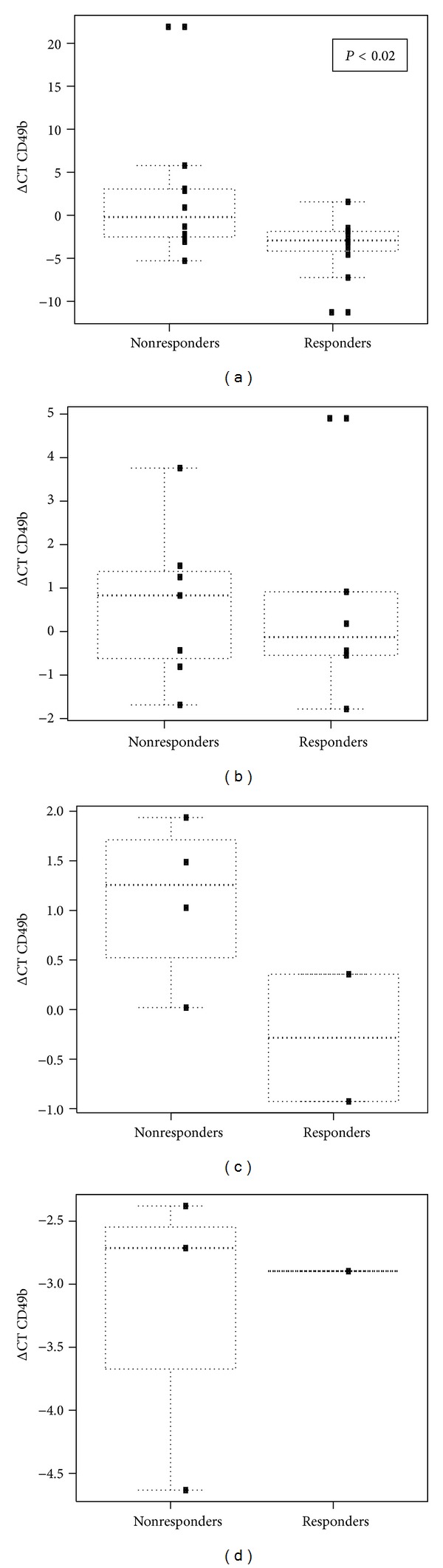
Relative expression of the CD49b marker using RT-PCR in responder and nonresponder groups. The responder group was defined by undetectable HCV RNA in peripheral blood, 18 and 30 months after beginning of the treatment (6 months after end of pegylated interferon + Ribavirin). (a) RT-PCR results obtained with Abi prism on sera from the patients. (b) RT-PCR results obtained with the light cycler in order to confirm Abi prism results on patient sera. (c) Results of RT-PCR on PBMC. (d) Results of RT-PCR on graft biopsies. Data are expressed as box plots in which the horizontal lines indicate the 25th, 50th, and 75th percentiles of the frequencies of the relative expression of CD49b, as measured by RT-PCR. The vertical lines represent the 10th and 90th percentiles.

By contrast, Tr1 levels, as assessed by the expression of the CD49b surface marker, were significantly associated with SVR. Indeed, at baseline, serum *CD49b* gene expression levels (relative expression: −2.93 CD49b [min; max: −11.29; 1.56]) were significantly lower in patients who would develop SVR when compared to non-responders (relative expression: −0.21 *CD49b* [min, max: −5.29; +21.9], *P* < 0.02) (Figure 2(a)).

Using an optimum cutoff of relative expression of −1.40, the sensitivity of CD49b <–1.40 to predict SVR was 0.917 (92%) (0.646–0.985), the specificity was 0.583 (58%) (0.320–0.807), and the area under rOC Curve was 0.771 (95% CI: 0.576–0.966).

The serum expression of other Tr1 markers did not differ significantly between SVR and non-SVR patients. Nevertheless, although it was not significant, the relative expression of *IL-10* and *CD18* gene expressions was lower in patients who would develop SVR +1.99, (95%, 0.27; 7.53) and +1.89 (95%, 0.31; 4.99) than in non-responders +3.61 ((–2.01; +8.91), *P* = 0.09) and +2.75 ((–1.28; +5.34), *P* = 0.008), respectively.

Results were confirmed by the light cycler-based PCR assay (Figure 2(b)).

### 3.3. Baseline Gene Expression of CD49b in PBMC

PBMCs were only available from ten of the study patients. As with serum transcriptional analysis, the results of PBMC mRNA analysis using the light cycler system revealed a lower *CD49b* gene expression in patients with SVR, but the difference was not significant (*P* = 0.06) (Figure 2(c)).

### 3.4. Baseline Gene Expression of CD49b in Liver Biopsy

Like the findings in serum and PBMC, the *CD49b* gene expression levels obtained in seven biopsy samples obtained from the study groups using the light cycler system were lower in the responder group than in the non-responder group (*P* = 0.07) (Figure 2(d)).

### 3.5. Peripheral Gene Expression of T Regulatory Markers at M12

The relative expressions of the same genes were also analyzed at 12 months (end of first course of antiviral therapy and time of randomization for the second course), and we looked for a correlation between the level of expression and the outcome of treatment, namely, SVR at 18 and 30 months. Using the ABI PRISM^R^ 7000 Sequence Detection System again, we found no association between the levels of all genes analyzed at 12 months and SVR at 18 and 30 months.

### 3.6. Cytometric Bead Array (CBA) at Baseline

Cytokines (IL-2, IL-4, IL-6, IL-10, TNF, IFN-*γ*, and IL-17A) were also assessed using the Cytometric Bead Array technology in sera from the 27 patients. None of these cytokine levels reached statistical significance regarding a prediction of the response to antiviral therapy; nevertheless, although it was not statistically significant, for IL-10, serum concentration was lower in patients who would develop SVR (15 ± 5 ng/mL) when compared to nonresponders (22 ± 12 ng/mL, *P* = 0.075) (data not shown).

## 4. Discussion 

We had recently observed that Treg markers were significantly enhanced in recurrent hepatitis C after LT and that Tr1 cell levels were specifically elevated in severe recurrent hepatitis C when compared to in mild recurrence [29]. The present study analyzed the potential correlation between regulatory T-cells, particularly Tr1, and the response to antiviral therapy after LT. This cohort study was extracted from a prospective clinical study designed to evaluate the treatment of recurrent HCV in liver transplant recipients. Serum samples were only available from 27 of the 101 patients included, but all data collected before, during, and after antiviral therapy were available for these patients. Initial experiments were performed on sera; this choice was deliberate although not optimum, because more serum samples than PBMC were available from the TRANSPEG patients. The data obtained with the ABI PRISM^R^ 7000 Sequence Detection System was validated using the light cycler-based PCR assay in sera, PBMC, and biopsies.

The findings of this study show for the first time that serum Tr1 gene expression levels, assessed from the expression of the surface marker CD49b before the introduction of antiviral therapy, were predictive of an SVR in patients with recurrent hepatitis C after LT. This result was confirmed in PBMC and liver biopsies obtained from the same patients, even if not statistically significant due to the poor number of samples. Patients with low Tr1 cell levels at baseline had a greater chance of a successful virological response than those with high Tr1 levels. Because HCV genotypes 1 and 4 are more resistant to antiviral therapy than 2 and 3, we checked that the predictive effect of CD49b on SVR was independent of the HCV genotype. It remains to be determined whether low Tr1 levels are constitutive or due to the patient's individual response to immunosuppressive therapy. Treg levels (CD4^+^CD25^+^Foxp3^+^) have already been evaluated during antiviral HCV therapy, and it has been suggested that they could predict the result of combination therapy [36]. We did not obtain this result in liver transplant recipients, probably because immunosuppressive therapy exerts a strong inhibitory effect on classical Tregs.

We hypothesize that Tr1 CD49b^+^ cells play a role in failure of the immune response during HCV recurrence after liver transplantation and may also participate in the failure of antiviral therapy. CD49b assessment in serum is predictive of the response to antiviral therapy. This new tool may help to select liver transplant recipients with HCV recurrence as candidates for more intensive antiviral therapy. This is an important finding because a recurrence of HCV infection is almost universal after LT, leading to accelerated disease when compared to that seen in immunocompetent patients [37] and to impaired patient and allograft survival [38], although some patients experience slow and progressive disease [35]. Moreover, pegylated interferon and ribavirin combination therapy in the transplant population is associated with serious adverse effects and a risk of rejection, with a sustained virological response rate reported as ranging from 26% to 48% [39, 40]. For this reason, antiviral therapy should be preferentially recommended in patients whose disease progression is highly predictive.

T-regulatory cells work, firstly, towards achieving tolerance of the graft [41] and secondly may enhance the severity of viral recurrence [42]. Our findings agree with these statements so that a patient with high Tr1 levels may appear to be at a greater risk of developing severe recurrent hepatitis C and with less chance of being treated effectively.

In the present study, *CD49b* was the only gene characterizing the Tr1 cell population as being associated significantly with the outcome of antiviral therapy. CD18, another specific marker for Tr1, was associated with CD49b in our previous study [29], but in this case CD18 was not associated with the outcome of antiviral therapy, even if we have obtained a non significant higher relative gene expression of *CD18* marker in non responder patients compared with responder ones. However, CD18 is a less specific marker of Tr1 than CD49b and it is expressed by numerous cell populations. In Tr1 cells, only a strong expression of CD18 is associated with CD49b expression [18, 34, 43]. Serum CD18 levels could have been influenced in different cell types by a variety of factors resulting in dissociation from CD49b, thus explaining why the difference between the low and high expression of *CD18* was not discriminately identified by PCR analysis. Although these data are encouraging, we acknowledge several limitations concerning the validation of CD49b expression level by complementary techniques. However, we can reasonably assume that the result obtained by RT-QPCR on serum RNA concerning CD49b level could be relevant in the context of the liver tissue. Indeed, it should be noted that a preliminary study from our team [29] has already established a closed correlation between the *CD49b* gene expression in the serum and the expression level of CD49b in the liver.

Moreover, because of the lack of numerous samples, it was not possible in this transcriptomic analysis to establish correlation between the different compartments studied, namely, serum, PBMC, and liver. However, we can assume that this correlation exists, since we published a previous study in which gene transcription was compared in 219 selected genes involved in the pathogenesis of HCV infection between sera, PBMCs, and liver samples collected simultaneously from five patients infected chronically. After amplification, significant correlations were observed between liver versus serum; liver versus PBMCs; and serum versus PBMCs [31]. In addition, a prospective trial is currently under way to confirm these data and to add functional assays, but the results will not be available for several years.

In conclusion, our results showed that the relative gene expression of *CD49b* was predictive of an SVR to treatment in the TRANSPEG study group. It is already well described in the literature that HCV genotypes 1 and 4 are more resistant to antiviral therapy than genotypes 2 and 3. We therefore verified whether this could influence our analysis, and if the difference shown was only due to responder genotypes, but in fact most of the responders were of genotypes 1 and 4. As expected, Tr1CD49b^+^ cells were predictive factors for a treatment response but there was no correlation with the HCV genotype.

Thus, Tr1 CD49b^+^ cells appear to play a role in failure of the immune response to HCV during its recurrence after LT and may also participate in the failure of antiviral therapy. Determining serum CD49b levels is predictive of the response to antiviral therapy. This new tool could be of value to select liver transplant recipients experiencing HCV recurrence as candidates for earlier and more intensive antiviral therapy. Further research is required to confirm these findings and particularly to verify whether CD49b is an independent predictive factor of response to antiviral therapy.

## Figures and Tables

**Table 1:** Primer sequence used in real-time RT-PCR.

Genes	Primers
*FOXP3 *	5′-TCACCTACGCCACGGTCAT-3′ 5′-CACAAAGCACTTGTGCAG-3′

*CD18 *	5′-ATGCTTGATGACCTCAGGAATG-3′ 5′-ACGGTCTTGTCCACGAAGGA-3′

*CD49b *	5′-CAACGGGTGTGTGTTCTGACA-3′ 5′-TCATCACACACAACCACAACATC-3′

*IL-10 *	5′-GAGAACCAAGACCAGACAT-3′ 5′-CCACGGCCTTGCTCTTGTT-3′

*IL-10 ra *	5′-CCGAGAGTATGAGATTGCCATTC-3′ 5′-CAGATGGTTTCACCTGGACACA-3′

*CD40 *	5′-TCCAGAACCACCCACTGCAT-3′ 5′-CACCGCAAGGAAGGCATT-3′

*CD40L *	5′-GAAAGAAAACAGCTTTGA-3′ 5′-TTTTTCAGCCCACTGTAACA-3′

*GITR *	5′-TGTGTCCAGCCTGAATTCCA-3′ 5′-CCGAGGCACAGTCGATACAC-3′

*CTLA-4 *	5′-TTCTTCTCTTCATCCCTGTCTTC-3′ 5′-GAGATGCATACTCACACACAAA-3′

*PD-1 *	5′-GCTACAACTGGGCTGGCG-3′ 5′-ATGTGTTGGAGAAGCTG-3′

*IL-2 *	5′-ACCAGGATGCTCACATTTAAGTTTTAC-3′ 5′-TCCAGAGGTTTGAGTTCTTCTTCTAGA-3′

*IFN-*γ**	5′-ATGTAGCGGATAATGGAACTC-3′ 5′-GACATTCAAGTCAGTTACC-3′

*TGF-*β**	5′-CGAGCCTGAGGCCGACTAC-3′ 5′-CGGAGCTCTGATGTGTTGAAGA-3′

*CD25 *	5′-GGGACTGCTCACGTTCATCA-3′ 5′-TTCAACATGGTTCCTTCCTTGTAG-3′

*CD4 *	5′-GGAAATCAGGGCTCCTTCT-3′ 5′-TGGTCCCAAAGGCTTCTTCTT-3′

*IL-4 *	5′-CACAAGCAGCTGATCCGATTC-3′ 5′-TCCAAGAAGTTTTCCAACGTA-3′

**β*-ACTINE *	5′-AGCCACACGCAGCTCATTG-3′ 5′-CACGGCATCGTCACCAACT-3′

**Table 2:** Demographic data of the cohort.

Characteristics	Distribution
Cohort study	27 patients
Inclusion date	August 2002 to January 2004
Time after transplant (year)*	3 (1, 8)
Age at selection*	56 (27, 68)
Gender	
Male	20
Female	7
HCV genotype**	Gen 1: *n* = 18 (67%), Gen 2: *n* = 1 (4%) Gen 3: *n* = 5 (18%), Gen 4: *n* = 3 (11%)
Immunosuppression	Tacrolimus: *n* = 16 (59%) Cyclosporine: *n* = 11 (41%)
Metavir *F* score at inclusion	*F*1:*n* = 15 *F*2: *n* = 8 *F*3: *n* = 3 *F*4: *n* = 1
Prothrombin time (%)	
Mean	90 ± 9
Median (min; max)	90 (59; 107)
AST/ALT (UI/L)	
(mean)	75 ± 45/100 ± 60
(median, min; max)	58 (24; 196)/82 (32; 293)
Bilirubin blood level at inclusion (*µ*mol/L)	15.7 ± 10.2
Viral load (log)	6.59 ± 0.24

*Median value (min, max)

**n = number of patients.

**Table 3:** Characteristics of responder and nonresponder groups at 18 months.

Characteristics	Responder (PCR < 0) *N* = 16	Nonresponder (PCR > 0) *N* = 11	*P*
% of patients	62%	38%	
Age (years)	54 ± 13	57 ± 7	0.53
Weight (kg)	74.7 ± 12.7	80.3 ± 14.3	0.39
HCV genotype	Gen 1: *n* = 10 (62%) Gen 2: *n* = 1 (6.5%) Gen 3: *n* = 4 (25%) Gen 4: *n* = 1 (6.5%)	Gen 1: *n* = 8 (73%) Gen 2: *n* = 0 Gen 3: *n* = 1 (9%) Gen 4: *n* = 2 (18%)	
Basal Creatinine (*µ*mol/L)	21.8 ± 24.9	103.3 ± 39.9	0.26
Cyclosporin/Tacrolimus	6 (46%)/7 (54%)	3 (38%)/5 (62%)	
Use of EPO	All patients	All patients	
Viral load (log)	6.22 ± 0.59	6.75 ± 0.42	0.54
METAVIR score (*n*)	*F*1 (10) *F*2 (5) *F*3 (1) *F*4 (0)	*F*1 (5) *F*2 (3) *F*3 (2) *F*4 (1)	
Donnor age > 40	53%	58%	NS

## Abbreviations

HCV: Hepatitis C virus
LT: Liver transplantation
Th1: T helper 1
Treg: regulatory T-cell
Tr1: Type 1 regulatory T-cell
SVR: Sustained virological response
PBMC: Peripheral blood Mononuclear cell
HCC: Hepatocellular carcinoma
CNI: Calcineurin inhibitors
Tac: Tacrolimus.

## References

[1] Negro F, Hadengue A (2006). Therapy of chronic hepatitis B. *Revue Medicale Suisse*.

[2] Kamal SM, Graham CS, He Q (2004). Kinetics of intrahepatic hepatitis C virus (HCV)—specific CD4^+^ T cell responses in HCV and Schistosoma mansoni coinfection: relation to progression of liver fibrosis. *Journal of Infectious Diseases*.

[3] Curry MP (2004). Hepatitis B and hepatitis C viruses in liver transplantation. *Transplantation*.

[4] Velidedeoglu E, Mange KC, Frank A (2004). Factors differentially correlated with the outcome of liver transplantation in HCV^+^ and HCV^−^ recipients. *Transplantation*.

[5] Berenguer M, Prieto M, San Juan F (2002). Contribution of donor age to the recent decrease in patient survival among HCV-infected liver transplant recipients. *Hepatology*.

[6] Thimme R, Oldach D, Chang K, Steiger C, Ray SC, Chisari FV (2001). Determinants of viral clearance and persistence during acute hepatitis C virus infection. *Journal of Experimental Medicine*.

[7] Gerlach JT, Diepolder HM, Jung M-C (1999). Recurrence of hepatitis C virus after loss of virus-specific CD4^+^ T-cell response in acute hepatitis C. *Gastroenterology*.

[8] Tsai S, Liaw Y, Chen M, Huang C, Kuo GC (1997). Detection of type 2-like T-helper cells in hepatitis C virus infection: implications for hepatitis C virus chronicity. *Hepatology*.

[9] Murata M, Nabeshima S, Maeda N, Nakashima H, Kashiwagi S, Hayashi J (2002). Increased frequency of IFN-*γ*-producing peripheral CD8^+^ T cells with memory-phenotype in patients with chronic hepatitis C. *Journal of Medical Virology*.

[10] O’Garra A, Vieira P (2004). Regulatory T cells and mechanisms of immune system control. *Nature Medicine*.

[11] Sakaguchi S, Sakaguchi N, Shimizu J (2001). Immunologic tolerance maintained by CD25^+^ CD4^+^ regulatory T cells: their common role in controlling autoimmunity, tumor immunity, and transplantation tolerance. *Immunological Reviews*.

[12] Sakaguchi S, Sakaguchi N, Asano M, Itoh M, Toda M (1995). Immunologic self-tolerance maintained by activated T cells expressing IL-2 receptor *α*-chains (CD25): breakdown of a single mechanism of self-tolerance causes various autoimmune diseases. *Journal of Immunology*.

[13] Shimizu J, Yamazaki S, Takahashi T, Ishida Y, Sakaguchi S (2002). Stimulation of CD25^+^CD4^+^ regulatory T cells through GITR breaks immunological self-tolerance. *Nature Immunology*.

[14] Birebent B, Lorho R, Lechartier H (2004). Suppressive properties of human CD4^+^CD25^+^ regulatory T cells are dependent on CTLA-4 expression. *European Journal of Immunology*.

[15] Hori S, Nomura T, Sakaguchi S (2003). Control of regulatory T cell development by the transcription factor Foxp3. *Science*.

[16] Morgan ME, van Bilsen JHM, Bakker AM (2005). Expression of FOXP3 mRNA is not confined to CD4^+^CD25^+^ T regulatory cells in humans. *Human Immunology*.

[17] Groux H, O’Garra A, Bigler M (1997). A CD4^+^ T-cell subset inhibits antigen-specific T-cell responses and prevents colitis. *Nature*.

[18] Rahmoun M, Foussat A, Groux H, Pène J, Yssel H, Chanez P (2006). Enhanced frequency of CD18- and CD49b-expressing T cells in peripheral blood of asthmatic patients correlates with disease severity. *International Archives of Allergy and Immunology*.

[19] Weiner HL (2001). Induction and mechanism of action of transforming growth factor-*β*-secreting Th3 regulatory cells. *Immunological Reviews*.

[20] Cabrera R, Tu Z, Xu Y (2004). An immunomodulatory role for CD4^+^CD25^+^ regulatory T lymphocytes in hepatitis C virus infection. *Hepatology*.

[21] Rushbrook SM, Ward SM, Unitt E (2005). Regulatory T cells suppress in vitro proliferation of virus-specific CD8+ T cells during persistent hepatitis C virus infection. *Journal of Virology*.

[22] Boettler T, Spangenberg HC, Neumann-Haefelin C (2005). T cells with a CD4^+^CD25^+^ regulatory phenotype suppress in vitro proliferation of virus-specific CD8^+^ T cells during chronic hepatitis C virus infection. *Journal of Virology*.

[23] MacDonald AJ, Duffy M, Brady MT (2002). CD4 T helper type 1 and regulatory T cells induced against the same epitopes on the core protein in hepatitis C virus-infected persons. *Journal of Infectious Diseases*.

[24] Velásquez-Lopera MM, Eaton VL, Lerret NM (2008). Induction of transplantation tolerance by allogeneic donor-derived CD4^+^CD25^+^Foxp3^+^ regulatory T cells. *Transplant Immunology*.

[25] Joffre O, Santolaria T, Calise D (2008). Prevention of acute and chronic allograft rejection with CD4^+^CD25^+^Foxp3^+^ regulatory T lymphocytes. *Nature Medicine*.

[26] Zeiser R, Nguyen VH, Beilhack A (2006). Inhibition of CD4^+^CD25^+^ regulatory T-cell function by calcineurin-dependent interleukin-2 production. *Blood*.

[27] Demirkiran A, Kok A, Kwekkeboom J (2006). Low circulating regulatory T-cell levels after acute rejection in liver transplantation. *Liver Transplantation*.

[28] Stenard F, Nguyen C, Cox K (2009). Decreases in circulating CD4^+^CD25^hi^FOXP3^+^ cells and increases in intragraft FOXP3^+^ cells accompany allograft rejection in pediatric liver allograft recipients. *Pediatric Transplantation*.

[29] Carpentier A, Conti F, Stenard F (2009). Increased expression of regulatory tr1 cells in recurrent hepatitis C after liver transplantation. *American Journal of Transplantation*.

[30] Calmus Y, Duvoux C, Pageaux G (2012). Treatment of recurrent HCV infection following liver transplantation: results of a multicenter, randomized, versus placebo, trial of ribavirin alone as maintenance therapy after one year of PegIFNalpha-2a plus ribavirin. *Journal of Hepatology*.

[31] Carpentier A, Conti F, Carrière M (2009). Analysis of gene transcription in sera during chronic hepatitis C infection. *Journal of Medical Virology*.

[32] Zhou X, Bailey-Bucktrout S, Jeker LT, Bluestone JA (2009). Plasticity of CD4^+^ FoxP3^+^ T cells. *Current Opinion in Immunology*.

[33] Banham AH (2006). Cell-surface IL-7 receptor expression facilitates the purification of FOXP3^+^ regulatory T cells. *Trends in Immunology*.

[34] Charbonnier L, Van Duivenvoorde LM, Apparailly F (2006). Immature dendritic cells suppress collagen-induced arthritis by in vivo expansion of CD49b^+^ regulatory T cells. *Journal of Immunology*.

[35] Firpi RJ, Abdelmalek MF, Soldevila-Pico C (2004). One-year protocol liver biopsy can stratify fibrosis progression in liver transplant recipients with recurrent hepatitis C infection. *Liver Transplantation*.

[36] Akiyama M, Ichikawa T, Miyaaki H (2010). Relationship between regulatory T cells and the combination of pegylated interferon and ribavirin for the treatment of chronic hepatitis type C. *Intervirology*.

[37] Feray C, Caccamo L, Alexander GJM (1999). European collaborative study on factors influencing outcome after liver transplantation for hepatitis C. *Gastroenterology*.

[38] Forman LM, Lewis JD, Berlin JA, Feldman HI, Lucey MR (2002). The association between hepatitis C infection and survival after orthotopic liver transplantation. *Gastroenterology*.

[39] Dumortier J, Scoazec J, Chevallier P, Boillot O (2004). Treatment of recurrent hepatitis C after liver transplantation: a pilot study of peginterferon alfa-2b and ribavirin combination. *Journal of Hepatology*.

[40] Rodriguez-Luna H, Khatib A, Sharma P (2004). Treatment of recurrent hepatitis C infection after liver transplantation with combination of pegylated interferon *α*2b and ribavirin: an open-label series. *Transplantation*.

[41] Manigold T, Racanelli V (2007). T-cell regulation by CD4 regulatory T cells during hepatitis B and C virus infections: facts and controversies. *The Lancet Infectious Diseases*.

[42] Smyk-Pearson S, Golden-Mason L, Klarquist J (2008). Functional suppression by FoxP3^+^CD4^+^CD25 high regulatory T cells during acute hepatitis C virus infection. *Journal of Infectious Diseases*.

[43] Charbonnier L, Han WGH, Quentin J (2009). Adoptive transfer of IL-10-secreting CD4^+^CD49b^+^ regulatory T cells suppresses ongoing arthritis. *Journal of Autoimmunity*.

